# Sleep, physical activity, and sedentary behaviors in relation to overall cancer and site-specific cancer risk: A prospective cohort study

**DOI:** 10.1016/j.isci.2024.109931

**Published:** 2024-05-09

**Authors:** Rongqi Zhang, Ying Lu, Zilong Bian, Siyun Zhou, Liying Xu, Fangyuan Jiang, Shuai Yuan, Xiao Tan, Xiangjun Chen, Yuan Ding, Xue Li

**Affiliations:** 1 Department of Big Data in Health Science School of Public Health and the Second Affiliated Hospital, Zhejiang University School of Medicine, Hangzhou, Zhejiang, China; 2 Institute of Environmental Medicine, Karolinska Institutet, Solna, Stockholm, Sweden; 3 Department of Big Data in Health Science, School of Public Health and Department of Psychiatry Sir Run Run Shaw Hospital, Zhejiang University School of Medicine, Hangzhou, Zhejiang, China; 4 Department of Medical Sciences, Uppsala University, Uppsala, Sweden; 5 Institute of Translational Medicine, Zhejiang University School of Medicine, 268 Kaixuan Road, Hangzhou 310020, China; 6 Department of Hepatobiliary and Pancreatic Surgery, the Second Affiliated Hospital, Zhejiang University School of Medicine, Hangzhou, Zhejiang, China; 7 Centre for Global Health, Usher Institute, University of Edinburgh, Edinburgh, UK

**Keywords:** Health sciences, Cancer

## Abstract

Large prospective studies are required to better elucidate the associations of physical activity, sedentary behaviors (SBs), and sleep with overall cancer and site-specific cancer risk, accounting for the interactions with genetic predisposition. The study included 360,271 individuals in UK Biobank. After a median follow-up of 12.52 years, we found higher total physical activity (TPA) level and higher sleep scores were related to reduced risk of cancer while higher SB level showed a positive association with cancer. Compared with high TPA-healthy sleep group and low SB-healthy sleep group, low TPA-poor sleep group and high SB-poor sleep group had the highest risk for overall cancer, breast cancer, and lung cancer. Adherence to a more active exercise pattern was associated with a lower risk of cancer irrespective of genetic risk. Our study suggests that improving the quality of sleep and developing physical activity habits might yield benefits in mitigating the cancer risk.

## Highlights

• Exploring joint impact of sleep, physical activity, sedentary behavior on cancer risk
• Integrating genetic predisposition into the analysis for a comprehensive approach
• Leveraging extensive individual-level data from the UKB to enhance statistical power

## Graphical abstract

**Figure:**
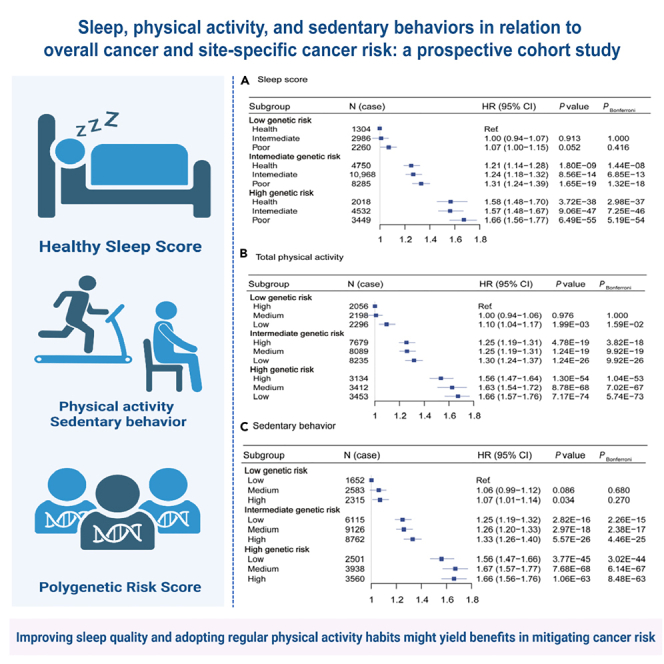

## Introduction

Cancer has been an important barrier to increasing life expectancy and the burden of cancer incidence is rapidly growing worldwide.^1^ In 2020, there were an estimated 19.3 million overall cancer cases worldwide and breast cancer is the most commonly diagnosed cancer (11.7% of total cases), closely followed by lung (11.4%), colorectal (10.0%), and prostate (7.3%).^2^ The main incidental cancers were prostate cancer in men and breast cancer in women.^3^ During the past decade, the number of incident cases and deaths from lung cancer increased globally.^4^ In 2018, cancers of the gastrointestinal tract accounted for more than one-quarter (26%) of the global cancer incidence, with colorectal cancer being the predominant malignant condition within this category (approximately 1.8 million new cases in 2018).^5^ Overall cancer and these four specific types of cancer exhibited significantly high rates of both incidence and mortality globally. The temporal changes in behavioral patterns may have partly contributed to the upward trend in cancer incidence and death.^6^ Behaviors are complex and their effects on cancer may depend on several different characteristics.^7^ Therefore, exploring the role of behavioral factors is critical for cancer prevention.

Physical activity (PA) is defined as the body’s large muscles moving rhythmically for a sustained period.^8^ Sedentary behavior (SB) is considered any waking behavior characterized by an energy expenditure ≤1.5 metabolic equivalent units (METs) while in a sitting or reclining posture, including television watching or computer use.^9^ Over the past decade, PA has been studied as a reasonable protective factor for cancer and exercise interventions have been suggested as beneficial for effectively reducing cancer risk and mortality.^10^^,^^11^^,^^12^^,^^13^ As some evidence suggests, sedentary time is associated with several health outcomes independently. For example, SB poses an increased risk of mortality and the development of cardiometabolic disease,^14^ particularly when accumulated in prolonged, uninterrupted bouts.^15^^,^^16^^,^^17^ However, there remains a lack of studies focusing on individuals who have both high PA level and long SB time. Large prospective studies are required to provide timely evidence and better elucidate the independent and joint associations of PA and SB and cancer risk.

In addition to PA and SB, sleep and circadian health represent a significant, yet understudied type of lifestyle factors.^18^ The health-related sleep characteristics include duration,^19^ quality,^20^^,^^21^ and daytime sleepiness.^22^ Emerging evidence has suggested that unhealthy sleep and circadian manifestations, including abnormal sleep duration, excessive daytime sleepiness, evening chronotype, and insomnia, are closely related to all-cause mortality, CVD risk, and cancer death.^23^^,^^24^^,^^25^ Given that these sleep behaviors are generally correlated and might interact mutually, the prospective study is warranted examining the association between combination of sleep behaviors and common cancer risk. Additionally, it is established that both genetic background and behavioral factors could contribute to cancer incident.^26^^,^^27^ However, whether the effects between behavioral factors and cancer risk may modified by the genetic susceptibility of cancer remain unknown.

In this study, we utilized individual-level data from the UK Biobank (UKB), the largest prospective cohort of community residents, to investigate the independent and joint associations of healthy sleep score, PA, and SB with common cancer risk and we further accounted for the interactions with genetic predisposition of overall cancer leveraging the genetic data.

## Results

The current study included 360,271 participants with available information on baseline characteristics, daily activities, and health status. The selection process of participants has been shown in Figure S1. Of participants, 41,397 (11.49%) developed overall cancer, 8644 (2.40%) developed prostate cancer, 7817 (2.17%) developed breast cancer, 4560 (1.27%) developed colorectal cancer, and 2765 (0.77%) developed lung cancer during a median follow-up of 12.52 years Table 1 summarizes basic characteristics of participants by overall cancer status. Participants were mostly white (88.30%) and aged (mean ± SD) 55.94 ± 8.11 years. About 5.07%, 5.47%, and 23.36% had diabetes, CVD, or hypertension at baseline, respectively. The majority were current drinkers (92.17%), and the average number of pack-years of smoking was approximately 20. At baseline, individuals with overall cancer were more likely to exhibit lower levels of TPA and higher levels of SB. (Table 1).

**Table 1:** Baseline characteristics of UK Biobank participants

Characteristic^a^	Total	Overall cancer status	
		Non-cancer	Cancer
Number of participants	360,271	318,874	41,397

**Sociodemographic**			

Age (years)	55.94 (8.11)	55.46 (8.12)	59.64 (7.00)
Sex (male)	161,939 (44.95)	140,347 (44.01)	21,592 (52.16)
Race (white)	318,110 (88.30)	280,802 (88.06)	37,308 (90.12)
Education (college and above)	118,857 (32.99)	106,650 (33.45)	12,207 (29.49)
Townsend Deprivation Index	−1.35 (3.06)	−1.36 (3.05)	−1.33 (3.10)
Height (m)	1.68 (0.09)	1.68 (0.09)	1.69 (0.09)
Body Mass Index (kg/m^2^)	27.43 (4.79)	27.37 (4.78)	27.85 (4.81)

**Medical factors**			

Colorectal cancer screening (yes)	106,031 (29.43)	90,687 (28.44)	15,344 (37.07)
Use of NSAIDs (yes)	162,682 (45.16)	142,892 (44.81)	19,790 (47.81)
Family history of cancer (yes)	94,670 (26.28)	82,060 (25.73)	12,610 (30.46)
Diabetes (yes)	18,280 (5.07)	15,458 (4.85)	2822 (6.82)
Cardiovascular diseases (yes)	19,706 (5.47)	16,297 (5.11)	3409 (8.23)
Hypertension (yes)	84,170 (23.36)	72,311 (22.68)	11,859 (28.65)

**Reproductive factors (female only)**			

Age at menarche (years)	12.60 (2.78)	12.60 (2.79)	12.58 (2.75)
Number of live births	1.83 (1.20)	1.83 (1.20)	1.85 (1.20)
Use of hormones (yes)	73,257 (20.33)	64,452 (20.21)	8805 (21.27)
Use of oral contraceptive (yes)	162,649 (45.15)	147,077 (46.12)	15,572 (37.62)

**Lifestyle factors**			

Smoke (pack-year)	20.23 (10.35)	19.93 (9.71)	22.55 (14.15)
Drinking status (current drinker)	332,067 (92.17)	293,961 (92.19)	38,106 (92.05)
Sleep duration (h/d)	7.15 (1.05)	7.14 (1.05)	7.19 (1.09)
Chronotype (early)	97,269 (27.00)	85,845 (26.92)	11,424 (27.60)
Insomnia (never/rarely)	88,844 (24.66)	79,261 (24.86)	9583 (23.15)
Snoring (no)	225,982 (62.73)	201,138 (63.08)	24,844 (60.01)
Daytime napping (never/rarely)	275,196 (76.39)	244,779 (76.76)	30,417 (73.48)
Total physical activity (high level) b	115,308 (32.01)	102,193 (32.05)	13,115 (31.68)
Sedentary behavior (low level) c	107,628 (29.87)	99,246 (31.12)	14,940 (36.09)

a Mean values (SD) for continuous variables and N (%) for categorical variables.

b Total physical activity was grouped as low level <971.000 MET min/week; medium level 971.000–2600.565 MET min/week; high level >2600.565 MET min/week.

c Sedentary behavior was grouped as low level <3 h/day; medium level 3–4 h/day; high level >4 h/day.

Table 2 shows the independent associations of sleep score, TPA, and SB with common cancer risk. After adjustment for potential covariates, higher sleep score was positively associated with lower cancer risk (for overall cancer, HR = 0.86, 95% CI: 0.83–0.90, for prostate cancer, HR = 0.90, 95%CI: 0.82–0.99, for breast cancer, HR = 0.76, 95%CI: 0.69–0.85, for colorectal cancer, HR = 0.86, 95%CI: 0.75–0.98 and for lung cancer, HR = 0.58, 95%CI: 0.49–0.69). When examined each sleep characteristic individually, excess sleep duration, evening chronotype, usually insomnia and snoring were all related to higher risk of overall cancer (Table S4). Compared with participants with high TPA level, those with low TPA level had 5% and 14% higher odds of overall cancer and lung cancer, respectively. Individuals with high SB level had 5%, 8% and 26% higher odds of overall cancer, colorectal cancer and lung cancer than those with low SB level, respectively (Table 2). We also conducted a series of sensitivity analyses and found that when additional adjusting the healthy diet score and psychological health status, excluding cases occurred in the first two years of follow-up, or excluding participants without any moderate-to-vigorous PA, the associations of TPA, SB, and sleep score with overall cancer risk remained statistically significant (Tables S5–S7). When examining these independent associations in subgroups with different smoke status, it was consistently observed that both poor sleep scores and low TPA levels were significantly associated with a higher risk of overall cancer, regardless of participants' smoking status. However, the associations between poor sleep scores, low TPA levels, high SB levels, and lung cancer risk were only evident among smokers (Table 3). Table 4 illustrates the HRs for various SB levels (with low SB level as reference) in subpopulations with different levels of TPA. Compared with high TPA level and low SB level combination, participants with low TPA level but also high SB level had higher incident risks for overall cancer and lung cancer (for overall cancer, HR = 1.07, 95%CI: 1.02–1.12; for lung cancer, HR = 1.33, 95%CI: 1.12–1.59). Furthermore, participants with high TPA level and high SB level combination also demonstrated increased odds of 3% for overall cancer and 36% for lung cancer. Table 5 presents the joint associations of TPA, SB and sleep scores with overall cancer risk as well as specific cancer types. In comparison to the high TPA-healthy sleep group, the low TPA-poor sleep group had the highest risk for overall cancer, breast cancer, and lung cancer. Similarly, compared with low SB-healthy sleep group, the high SB-poor sleep group had 10%, 13%, and 39% increased odds of developing overall cancer, breast cancer, and lung cancer, respectively.

**Table 2:** The independent associations of exposures with overall cancer and specific cancer risk (*n* = 360,271)

Activity^d^	Overall cancer risk^a^			Specific cancer risk											
				Prostate cancer			Breast cancer^b^			Colorectal cancer^c^			Lung cancer		
	N (case)	HR (95%CI)	*p* value	N (case)	HR (95%CI)	*p* value	N (case)	HR (95%CI)	*p* value	N (case)	HR (95%CI)	*p* value	N (case)	HR (95%CI)	*p* value
**Sleep score**															

Healthy (74,290)	8234	Ref.		1632	Ref.		1568	Ref.		917	Ref.		523	Ref.	
Intermediate (164,899)	18,845	1.02 (0.99–1.04)	0.242	4180	1.06 (1.00–1.12)	**4.48E-02**	3496	1.03 (0.97–1.10)	0.294	2105	1.01 (0.93–1.09)	0.829	1118	1.03 (0.93–1.15)	0.548
Poor (121,082)	14,318	1.07 (1.04–1.10)	**8.03E-07**	2832	1.08 (1.01–1.14)	**1.85E-02**	2753	1.12 (1.05–1.20)	**3.09E-04**	1538	1.04 (0.96–1.13)	0.308	1124	1.32 (1.19–1.47)	**1.52E-07**
Per score increased	41,397	0.86 (0.83–0.90)	**2.70E-10**	8644	0.90 (0.82–0.99)	**3.24E-02**	7817	0.76 (0.69–0.85)	**5.15E-07**	4560	0.86 (0.75–0.98)	**2.69E-02**	2765	0.58 (0.49–0.69)	**2.55E-10**

**Total physical activity**															

High (*n* = 115,308)	13,115	Ref.		3036	Ref.		2169	Ref.		1442	Ref.		859	Ref.	
Medium (*n* = 124,912)	13,992	1.01 (0.99–1.03)	0.435	2963	1.01 (0.96–1.06)	0.711	2801	1.02 (0.91–1.13)	0.766	1541	1.02 (0.95–1.09)	0.632	827	0.94 (0.85–1.03)	0.207
Low (*n* = 120,051)	14,290	1.05 (1.03–1.08)	**7.33E-05**	2645	1.00 (0.95–1.05)	0.936	2847	1.03 (0.92–1.15)	0.597	1577	1.07 (0.99–1.15)	0.071	1079	1.14 (1.04–1.25)	**4.08E-03**

**Sedentary behavior**															

Low (*n* = 107,628)	10,480	Ref.		1994	Ref.		2465	Ref.		1127	Ref.		560	Ref.	
Medium (*n* = 138,457)	15,977	1.03 (1.00–1.05)	**3.26E-02**	3437	1.03 (0.97–1.09)	0.303	3043	1.03 (0.97–1.09)	0.321	1753	1.03 (0.95–1.11)	0.459	985	1.07 (0.96–1.19)	0.217
High (*n* = 114,186)	14,940	1.05 (1.03–1.08)	**7.49E-05**	3213	1.01 (0.95–1.07)	0.763	2309	1.01 (0.96–1.08)	0.643	1680	1.08 (1.00–1.17)	**4.54E-02**	1220	1.26 (1.13–1.40)	**1.70E-05**

The value in bold denotes significant differences (*p* < 0.05).

a Adjusted by age at recruitment (continuous), sex (male/female), ethnicity (white/non-white/unknown), education (college/high school and below/unknown), TDI (continuous), smoke (pack-year), alcohol intake (never/former/current), height (continuous), body mass index (continuous), use of NSAIDs (yes/no), family history of cancer (yes/no), diabetes (yes/no), hypertension (yes/no), CVD (yes/no). Sleep score, total physical activity and sedentary behavior mutually adjusted.

b Additionally adjusted by age at menarche (continuous), number of live birth (continuous), use of hormones (yes/no) and use of oral contraceptive (yes/no).

c Additionally adjusted by colorectal cancer screening (yes/no).

d Sleep scores were categorized into: poor, T1; intermediate, T2; healthy, T3. Total physical activity was grouped as low level <971.000 MET min/week; medium level 971.000–2600.565 MET min/week; high level >2600.565 MET min/week. Sedentary time was grouped as low level <3 h/day; medium level 3–4 h/day; high level >4 h/day.

**Table 3:** Associations of sedentary behavior with overall cancer and specific cancer risk in subpopulations with different levels of total physical activity

Activity^a^	High level of TPA (*N* = 115,308)			Medium level of TPA (*N* = 124,912)			Low level of TPA (*N* = 120,051)			*P* interaction^e^
	N (case)	HR (95%CI)	*p* value	N (case)	HR (95%CI)	*p* value	N (case)	HR (95%CI)	*p* value	
**Overall cancer**^b^										

Sedentary behavior										0.574
Low	3,515	Ref.		3,728	Ref.		3,237	Ref.		
Medium	5,314	1.01 (0.97–1.06)	0.529	5,505	1.04 (0.99–1.08)	0.096	5,158	1.04 (0.99–1.08)	0.103	
High	4,286	1.08 (1.03–1.13)	**1.33E-03**	4,759	1.03 (0.99–1.08)	0.141	5,895	1.07 (1.02–1.12)	**4.10E-03**	

**Prostate cancer**										

Sedentary behavior										0.464
Low	732	Ref.		721	Ref.		541	Ref.		
Medium	1,294	1.04 (0.95–1.14)	0.367	1,189	1.02 (0.93–1.12)	0.647	954	1.03 (0.93–1.15)	0.579	
High	1,010	1.03 (0.94–1.14)	0.533	1,053	0.97 (0.88–1.07)	0.562	1,150	1.04 (0.94–1.16)	0.456	

**Breast cancer**^c^										

Sedentary behavior										0.840
Low	722	Ref.		918	Ref.		825	Ref.		
Medium	866	1.01 (0.91–1.12)	0.851	1,094	1.04 (0.95–1.14)	0.408	1,083	1.03 (0.94–1.13)	0.484	
High	581	1.02 (0.91–1.14)	0.772	789	1.04 (0.94–1.15)	0.464	939	1.01 (0.92–1.12)	0.792	

**Colorectal cancer**^d^										

Sedentary behavior										0.508
Low	382	Ref.		384	Ref.		361	Ref.		
Medium	591	1.02 (0.89–1.16)	0.805	589	1.05 (0.92–1.19)	0.488	573	1.02 (0.90–1.17)	0.722	
High	469	1.07 (0.93–1.23)	0.355	568	1.15 (1.01–1.32)	0.037	643	1.03 (0.90–1.18)	0.621	

**Lung cancer**										

Sedentary behavior										0.863
Low	194	Ref.		181	Ref.		185	Ref.		
Medium	332	1.10 (0.92–1.32)	0.296	321	1.09 (0.91–1.31)	0.358	332	1.05 (0.88–1.26)	0.585	
High	333	1.36 (1.14–1.63)	**8.56E-04**	325	1.16 (0.96–1.40)	0.133	562	1.33 (1.12–1.59)	**1.20E-03**	

The value in bold denotes significant differences (*p* < 0.05).

a Total physical activity was grouped as low level <971.000 MET min/week; medium level 971.000–2600.565 MET min/week; high level >2600.565 MET min/week. Sedentary time was grouped as low level <3 h/day; medium level 3–4 h/day; high level >4 h/day.

b Adjusted by age at recruitment (continuous), sex (male/female), ethnicity (white/non-white/unknown), education (college/high school and below/unknown), TDI (continuous), smoke (pack-year), alcohol intake (never/former/current), height (continuous), body mass index (continuous), use of NSAIDs (yes/no), family history of cancer (yes/no), diabetes (yes/no), hypertension (yes/no), CVD (yes/no).

c Additionally adjusted by age at menarche (continuous), number of live birth (continuous), use of hormones (yes/no) and use of oral contraceptive (yes/no).

d Additionally adjusted by colorectal cancer screening (yes/no).

e The p-interaction was tested by log likelihood ratio (LR-) tests comparing a model with the interaction term of sedentary time (h/d) and total physical activity (MET min/week) to a model without.

**Table 4:** The joint associations of activities and sleep scores with overall cancer and specific cancer risk

Activity^a^	Healthy sleep score (*N* = 74,290)				Intermediate sleep score (*N* = 164,899)				Poor sleep score (*N* = 121,082)				Interaction *p* value^e^
	N (case)	HR (95%CI)	*p* value	*P*_Bonferroni_	N (case)	HR (95%CI)	*p* value	*P*_Bonferroni_	N (case)	HR (95%CI)	*p* value	*P*_Bonferroni_	
**Overall cancer risk**^b^													

Total physical activity													0.792
High	2,961	Ref.			6,219	1.01 (0.97–1.06)	0.654	1.000	3,935	1.06 (1.01–1.11)	**1.79E-02**	0.143	
Medium	2,717	0.98 (0.93–1.04)	0.523	1.000	6,507	1.02 (0.98–1.07)	0.322	1.000	4,768	1.09 (1.04–1.14)	**3.45E-04**	**2.76E-03**	
Low	2,556	1.06 (1.01–1.12)	**2.19E-02**	0.175	6,119	1.06 (1.02–1.11)	**8.12E-03**	0.065	5,615	1.12 (1.07–1.17)	**1.46E-06**	**1.17E-05**	
Sedentary behavior													0.097
Low	2,330	Ref.			4,869	1.09 (1.03–1.14)	**7.67E-04**	**6.14E-03**	3,281	1.13 (1.08–1.19)	**9.16E-07**	**7.33E-06**	
Medium	3,245	1.04 (0.98–1.10)	0.202	1.000	7,496	1.04 (0.99–1.09)	0.098	0.784	5,236	1.10 (1.05–1.15)	**1.62E-04**	**7.33E-06**	
High	2,659	1.04 (0.99–1.10)	0.112	0.896	6,480	1.01 (0.96–1.06)	0.749	1.000	5,801	1.08 (1.03–1.14)	**2.84E-03**	**2.27E-02**	

**Prostate cancer risk**													

Total physical activity													**4.61E-02**
High	611	Ref.			1,515	1.11 (1.01–1.22)	**3.29E-02**	0.263	910	1.18 (1.06–1.30)	**1.96E-03**	**1.57E-02**	
Medium	555	1.06 (0.943–1.19)	0.333	1.000	1,426	1.10 (1.00–1.21)	**4.53E-02**	0.362	982	1.17 (1.06–1.29)	**2.42E-03**	**1.94E-02**	
Low	466	1.12 (0.99–1.26)	0.071	0.568	1,239	1.14 (1.03–1.26)	**8.92E-03**	0.071	940	1.05 (0.95–1.17)	0.320	1.000	
Sedentary behavior													0.164
Low	433	Ref.			998	1.06 (0.95–1.19)	0.289	1.000	563	1.01 (0.89–1.15)	0.861	1.000	
Medium	630	0.98 (0.87–1.11)	0.791	1.000	1,708	1.07 (0.96–1.19)	0.232	1.000	1,099	1.12 (1.00–1.25)	**4.75E-02**	0.380	
High	569	1.01 (0.90–1.15)	0.819	1.000	1,474	1.05 (0.94–1.17)	0.400	1.000	1,170	1.06 (0.95–1.19)	0.272	1.000	

**Breast cancer risk**^c^													

otal physical activity													0.892
igh	514	Ref.			997	0.99 (0.89–1.10)	0.799	1.000	658	1.06 (0.94–1.19)	0.335	1.000	
Medium	550	1.02 (0.90–1.15)	0.791	1.000	1,285	1.09 (0.99–1.21)	0.093	0.744	966	1.19 (1.07–1.32)	**1.83E-03**	**1.46E-02**	
Low	504	1.07 (0.95–1.21)	0.265	1.000	1,214	1.11 (1.00–1.23)	**4.70E-02**	0.376	1,129	1.22 (1.10–1.35)	**2.37E-04**	**1.90E-03**	
Sedentary behavior													0.549
Low	554	Ref.			1,135	1.00 (0.91–1.11)	0.964	1.000	776	1.10 (0.99–1.23)	0.089	0.712	
Medium	623	1.03 (0.92–1.16)	0.599	1.000	1,379	1.03 (0.93–1.14)	0.569	1.000	1,041	1.14 (1.02–1.26)	**1.58E-02**	0.126	
High	391	0.93 (0.81–1.06)	0.267	1.000	982	1.06 (0.95–1.18)	0.274	1.000	936	1.13 (1.02–1.26)	**2.32E-02**	0.186	

**Colorectal cancer risk**^d^													

otal physical activity													0.725
gh	342	Ref.			679	0.95 (0.83–1.08)	0.407	1.000	421	0.99 (0.86–1.14)	0.913	1.000	
Medium	291	0.92 (0.79–1.07)	0.281	1.000	725	0.98 (0.87–1.12)	0.810	1.000	525	1.06 (0.92–1.21)	0.437	1.000	
Low	284	1.04 (0.89–1.22)	0.591	1.000	701	1.06 (0.93–1.21)	0.392	1.000	592	1.05 (0.92–1.20)	0.508	1.000	
Sedentary behavior													0.496
Low	251	Ref.			511	0.98 (0.84–1.14)	0.752	1.000	365	1.13 (0.96–1.33)	0.132	1.000	
Medium	383	1.12 (0.96–1.32)	0.154	1.000	836	1.05 (0.91–1.21)	0.517	1.000	534	1.03 (0.89–1.20)	0.657	1.000	
High	283	1.01 (0.85–1.20)	0.935	1.000	758	1.14 (0.99–1.32)	0.068	0.544	639	1.15 (0.99–1.33)	0.064	0.512	

**Lung cancer risk**													

Total physical activity													0.420
High	191	Ref.			384	1.05 (0.88–1.25)	0.599	1.000	284	1.23 (1.02–1.48)	**2.63E-02**	0.210	
Medium	159	0.94 (0.76–1.16)	0.586	1.000	336	0.93 (0.78–1.11)	0.418	1.000	332	1.26 (1.05–1.50)	**1.17E-02**	0.094	
Low	173	1.09 (0.89–1.34)	0.393	1.000	398	1.17 (0.98–1.39)	0.075	0.600	508	1.53 (1.30–1.81)	**5.97E-07**	**4.78E-06**	
Sedentary behavior													0.291
Low	116	Ref.			241	1.1 (0.885–1.38)	0.378	1.000	203	1.44 (1.14–1.8)	**1.91E-03**	**1.53E-02**	
Medium	201	1.2 (0.954–1.51)	0.119	0.952	397	1.09 (0.884–1.34)	0.428	1.000	387	1.58 (1.29–1.95)	**1.50E-05**	**1.20E-04**	
High	206	1.31 (1.04–1.65)	**2.05E-02**	0.164	480	1.47 (1.2–1.81)	**2.09E-04**	**1.67E-03**	534	1.68 (1.37–2.06)	**4.96E-07**	**3.97E-06**	

The value in bold denotes significant differences (*p* < 0.05).

a Total physical activity was grouped as low level <971.000 MET min/week; medium level 971.000–2600.565 MET min/week; high level >2600.565 MET min/week. Sedentary time was grouped as low level <3 h/day; medium level 3–4 h/day; high level >4 h/day.

b Adjusted by age at recruitment (continuous), sex (male/female), ethnicity (white/non-white/unknown), education (college/high school and below/unknown), TDI (continuous), smoke (pack-year), alcohol intake (never/former/current), height (continuous), body mass index (continuous), use of NSAIDs (yes/no), family history of cancer (yes/no), diabetes (yes/no), hypertension (yes/no), CVD (yes/no).

c Additionally adjusted by age at menarche (continuous), number of live birth (continuous), use of hormones (yes/no) and use of oral contraceptive (yes/no).

d Additionally adjusted by colorectal cancer screening (yes/no).

e The p-interaction was tested by log likelihood ratio (LR-) tests comparing a model with the interaction term (i.e., 6-level categorical variable of categories of sleep score combined with categories of total physical activity and in turn sedentary behavior) to a model without (i.e., separate 3-level categorical variables of sleep score, total physical activity, and sedentary behavior).

**Table 5:** The associations of sedentary behavior, physical activity, and sleep with overall cancer and specific cancer risk in subpopulations with different smoke status

Activity^a^	Smoker			Non-smoker			Interaction *p* value^e^
	N (case)	HR (95%CI)	*p* value	N (case)	HR (95%CI)	*p* value	
**Overall cancer risk**^b^							

Sleep score							0.517
High	4,097	Ref.		4,137	Ref.		
Medium	9,688	1.00 (0.96–1.04)	0.989	9,157	1.02 (0.98–1.06)	0.323	
Low	8,416	1.06 (1.02–1.10)	**3.46E-03**	5,902	1.06 (1.02–1.11)	**2.18E-03**	
Total physical activity							0.219
High	7,111	Ref.		6,004	Ref.		
Medium	7,342	1.03 (0.99–1.07)	0.201	6,650	1.00 (0.97–1.03)	0.968	
Low	7,748	1.08 (1.03–1.13)	**2.26E-03**	6,542	1.05 (1.01–1.08)	**1.46E-02**	
Sedentary behavior							0.412
Low	4,770	Ref.		5,710	Ref.		
Medium	8,389	1.04 (1.00–1.07)	0.051	7,588	1.01 (0.98–1.05)	0.417	
High	9,042	1.10 (1.06–1.14)	**4.15E-07**	5,898	1.00 (0.97–1.04)	0.885	

**Prostate cancer risk**							

Sleep score							0.760
High	794	Ref.		838	Ref.		
Medium	2,105	1.03 (0.95–1.12)	0.434	2,075	1.09 (1.01–1.18)	**3.13E-02**	
Low	1,591	1.05 (0.96–1.14)	0.305	1,241	1.12 (1.03–1.22)	**1.17E-02**	
Total physical activity							0.194
High	1,605	Ref.		1,431	Ref.		
Medium	1,496	1.01 (0.94–1.08)	0.837	1,467	1.01 (0.94–1.09)	0.775	
Low	1,389	0.99 (0.92–1.06)	0.727	1,256	1.01 (0.93–1.09)	0.833	
Sedentary behavior							0.391
Low	911	Ref.		1,083	Ref.		
Medium	1,759	1.04 (0.96–1.12)	0.372	1,678	1.03 (0.95–1.11)	0.518	
High	1,820	1.03 (0.95–1.12)	0.479	1,393	0.99 (0.92–1.08)	0.874	

**Breast cancer risk**^c^							

Sleep score							0.693
High	620	Ref.		948	Ref.		
Medium	1,429	1.02 (0.93–1.12)	0.664	2,067	1.04 (0.96–1.12)	0.349	
Low	1,289	1.06 (0.97–1.17)	0.212	1,464	1.16 (1.07–1.26)	**3.94E-04**	
Total physical activity							0.783
High	959	Ref.		1,210	Ref.		
Medium	1,205	1.07 (0.98–1.16)	0.123	1,596	1.11 (1.03–1.19)	**8.64E-03**	
Low	1,174	1.04 (0.95–1.13)	0.428	1,673	1.18 (1.10–1.28)	**1.08E-05**	
Sedentary behavior							0.451
Low	912	Ref.		1,553	Ref.		
Medium	1,276	1.02 (0.93–1.11)	0.690	1,767	1.04 (0.97–1.11)	0.333	
High	1,150	1.07 (0.98–1.17)	0.151	1,159	0.97 (0.89–1.05)	0.427	

**Colorectal cancer risk**^d^							

Sleep score							0.249
High	460	Ref.		457	Ref.		
Medium	1,060	0.96 (0.86–1.07)	0.406	1,045	1.05 (0.94–1.18)	0.364	
Low	929	1.04 (0.93–1.17)	0.474	609	1.01 (0.90–1.14)	0.851	
Total physical activity							0.273
High	769	Ref.		673	Ref.		
Medium	827	1.06 (0.96–1.17)	0.241	714	0.97 (0.87–1.08)	0.597	
Low	853	1.09 (0.98–1.20)	0.104	724	1.06 (0.95–1.18)	0.272	
Sedentary behavior							0.982
Low	536	Ref.		591	Ref.		
Medium	920	0.99 (0.89–1.11)	0.891	833	1.06 (0.95–1.18)	0.278	
High	993	1.06 (0.95–1.18)	0.334	687	1.11 (0.99–1.24)	0.079	

**Lung cancer risk**							

Sleep score							0.407
High	444	Ref.		79	Ref.		
Medium	913	0.95 (0.85–1.07)	0.401	205	1.24 (0.96–1.61)	0.103	
Low	1,005	1.23 (1.10–1.37)	**3.92E-04**	119	1.18 (0.88–1.57)	0.263	
Total physical activity							0.941
High	731	Ref.		128	Ref.		
Medium	701	0.96 (0.86–1.06)	0.431	126	0.89 (0.69–1.14)	0.346	
Low	930	1.20 (1.09–1.33)	**2.38E-04**	149	1.13 (0.89–1.43)	0.333	
Sedentary behavior							0.150
Low	433	Ref.		127	Ref.		
Medium	835	1.07 (0.95–1.20)	0.290	150	0.92 (0.73–1.17)	0.517	
High	1,094	1.28 (1.14–1.44)	**2.23E-05**	126	1.00 (0.78–1.30)	0.974	

The value in bold denotes significant differences (*p* < 0.05).

a Sleep scores were categorized into: poor, T1; intermediate, T2; healthy, T3. Total physical activity was grouped as low level <971.000 MET min/week; medium level 971.000–2600.565 MET min/week; high level >2600.565 MET min/week. Sedentary time was grouped as low level <3 h/day; medium level 3–4 h/day; high level >4 h/day.

b Adjusted by age at recruitment (continuous), sex (male/female), ethnicity (white/non-white/unknown), education (college/high school and below/unknown), TDI (continuous), alcohol intake (never/former/current), height (continuous), body mass index (continuous), use of NSAIDs (yes/no), family history of cancer (yes/no), diabetes (yes/no), hypertension (yes/no), CVD (yes/no), sleep score, total physical activity and sedentary behavior mutually adjusted.

c Additionally adjusted by age at menarche (continuous), number of live birth (continuous), use of hormones (yes/no) and use of oral contraceptive (yes/no).

d Additionally adjusted by colorectal cancer screening (yes/no).

e The p-interaction was tested by log likelihood ratio (LR-) tests comparing a model with the interaction term of sedentary time (h/d) and total physical activity (MET min/week) to a model without.

**Figure 1:**
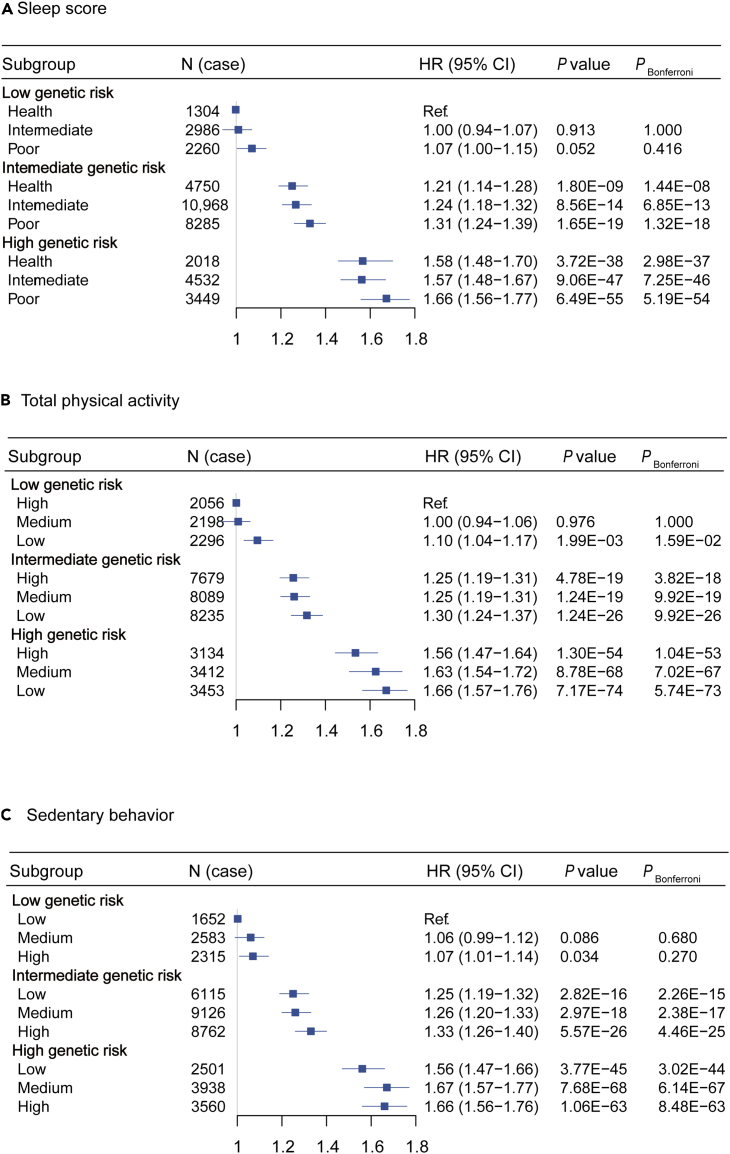
Risk of incident overall cancer by joint categorization for genetic risk and healthy sleep score, physical activity, and sedentary behavior 1. (A) Sleep score, *P* interaction = 0.782; (B) total physical activity, *P* interaction = 0.589; (C) sedentary behavior, *P* interaction = 0.699. 2. Adjusted by age at recruitment age (continuous), sex (male/female), ethnicity (white/non-white/unknown), education (college/high school and below/unknown), TDI (continuous), smoke (pack-year), alcohol intake (never/former/current), height (continuous), body mass index (continuous), use of NSAIDs (yes/no), family history of cancer (yes/no), diabetes (yes/no), hypertension (yes/no), CVD (yes/no). Sleep score, total physical activity and sedentary behavior mutually adjusted. 3. Participants were then categorized into low genetic risk (Q1), intermediate genetic risk (Q2-4), and high genetic risk (Q5) according to overall cancer genetic risk score. 4. Sleep scores were categorized into: poor, T1; intermediate, T2; healthy, T3. Total physical activity was grouped as low level <971.000 MET min/week; medium level 971.000–2600.565 MET min/week; high level >2600.565 MET min/week. Sedentary behavior was grouped as low level <3 h/day; medium level 3–4 h/day; high level >4 h/day.

As shown in Figure 1, the overall cancer risks increased with both genetic risk and poor sleep, lower TPA level or higher SB level preference in a dose–response manner. Compared with individuals with low genetic risk and healthy sleep score, 66% higher cancer risk were observed in those with high genetic risk and poor sleep score (Figure 1A). Similarly, high genetic risk-low TPA level group as well as high genetic risk-high SB level group was associated with 66% higher cancer risk (Figures 1B and 1C). Furthermore, we did not detect any multiplicative interaction between the genetic risk and the sleep score, TPA or SB (*p* = 0.782 for sleep score, *p* = 0.589 for TPA, and *p* = 0.699 for SB).

## Discussion

We leveraged a large population-based cohort study using UKB data and systematically investigated the independent and joint associations across sleep score, PA, SB, and overall cancer and site-specific cancer risk, further considering the interactions with genetic predisposition of overall cancer. We observed a significantly increased cancer risk among participants with poor sleep score, low TPA level, or high SB level. Compared with high PA-healthy sleep group and low SB- healthy sleep group, low PA-poor sleep group and high SB-poor sleep group had the highest risk for overall cancer, breast cancer, and lung cancer. Those with high genetic risk and low level of TPA had the greatest incident risk of overall cancer, compared with those who had low genetic risk and high TPA level and adherence to a healthier activity pattern might diminish the cancer risk among individuals regardless of genetic background.

According to the previous epidemiological studies, each sleep trait had been reported to be associated with cancer risk, for instance, sleep duration had a U-shape association with lung and colorectal cancer risk^28^^,^^29^ and per 1-h increment of sleep was identified had a negative correlation with endometrial cancer risk among obese women,^30^ morning chronotype might associated with a decreased risk of overall digestive tract cancer,^31^ insomnia was found to exhibit positive correlation between insomnia and malignant neoplasm of the liver and intrahepatic bile ducts,^32^ as well as participants who regularly snored and reported longer sleep time demonstrated a heightened susceptibility to colorectal cancer.^33^ Evaluating the combination of these sleep behaviors is crucial due to their frequent interconnections and existing evidence supports selecting these five sleep traits in the establishment of the comprehensive sleep scores. In line with our findings, the work^34^ by Chen et al. also highlighted that individuals adhered healthier sleep pattern had lower risk of colorectal cancer incidence. Our study extended the findings from the above study by capturing different perspectives of sleep behaviors and different site-specific cancers. Our finding as well as previous evidence moreover backed the positive relationship between healthy sleep pattern and cancer prevention and underlined the importance of considering sleep behaviors in cancer prevention practices. In addition, the healthy definition of each sleep behavior and the methodology for establishing a healthy sleep score in our study provided a useful reference in facilitating sleep hygiene strategies. In current study, we systematically created site-specific PRSs for 17 cancer types, and constructed overall PRSs. Participants with a high genetic risk and poor sleep score had the greatest incident overall cancer risk, compared with those with a low genetic risk and healthy sleep score. Additionally, we further investigated the potential interaction between sleep score and genetic predisposition to overall cancer but no statistically significant findings were observed. Whereas, our finding still supported that improving the sleep pattern could reduce overall cancer risk within each genetic risk group. The assessment of genetic risk scores could encourage people to participate in precision prevention strategies. Although characteristics of gene were unchangeable, effective behavioral interventions for individuals at a high genetic risk were still to be effective precautions for cancer prevention. Further investigations are warranted to elucidate the underlying mechanism through which the combination between sleep behaviors and genetic predisposition may impact cancer susceptibility, as well as to comprehend the synergistic effect of these risk factors on augmenting cancer incidence.

In present study, we found that both low TPA level and high SB level was associated with an increased overall risk of cancer independently. Furthermore, even among individuals engaging in adequate physical exercise, prolonged periods of SB significantly elevated overall cancer risk. The interaction between TPA and SB was non-significant in our study. Nevertheless, previous evidence^35^ has suggested that high PA levels could alter the relationship between SB and overall cancer risk. Further studies are warranted to investigate the potential mechanisms underlying the association between TPA and SB and cancer risk. Meanwhile, we examined the association between TPA or SB and overall cancer risk across genetic risk groups. We found that participants with high genetic risk and low TPA level showed the highest overall cancer risk compared to those low genetic risk and high TPA level. Participants with high genetic risk and high SB level showed a significantly elevated incidence risk of cancer, in comparison to those with low genetic risk and low SB level. Adhering to favorable physical exercises and reducing SB could provide benefits irrespective of genetic risk groups. The potential multiplicative interaction between TPA or SB and genetic predisposition for overall cancer was also investigated in our study, however, no statistically significant interaction was observed.

In comparison to individuals with high TPA level combined with healthy sleep scores, low TPA level group showed significantly increased cancer risks irrespective of sleep score. A recent study^36^ by Huang et al. indicated that poor sleep score was associated with higher risk for cancer mortality, particularly among participants with insufficient TPA. Existing evidence supported that sleep and PA were not independent activities but may interact with each other.^37^ Notably, our study revealed a synergistic interaction between sleep score and TPA level in relation to the risk of prostate cancer, suggesting that adherence to favorable physical exercises and healthy sleep pattern would be helpful in prostate cancer prevention.

### Limitations of the study

The present study has several strengths, including the large sample size, the joint analysis of the healthy sleep score, PA, and SB to gain a convincing understanding on cancer risk and the prospective study design with extensive measurement of covariates, which allowed us to strengthen our interpretation. Our study also has some potential limitations. First, we chose five common sleep characteristics to conduct the healthy sleep score and did not include all the sleep traits, such as rapid eye movement (REM) sleep, bed-sharing/co-sleeping, and restless legs syndrome. Future studies that incorporate more sleep behaviors into the healthy sleep score could be more robust and credible. Second, even though we had adjusted for main confounding factors, residual confounding from unknown or unmeasured factors probably remains. Future studies could consider more comprehensive confounding factors, such as adverse childhood experiences and violence related factors, if data available. Third, the baseline information in our study was based on self-reported questionnaires and the findings might suffer from self-report bias. Future studies based on wearable devices that measure sleep or PA will fill this limitation. Finally, the present study was based on UKB and most participants were middle-aged and older European. This might affect the generalization of the results to other populations but not affect the internal validity of the study. Future studies are warranted to re-evaluate the associations between sleep score, PA, and cancer risk among younger multiethnic population.

### Conclusions

This is the first and the largest study addressing the independent and joint associations of SB, TPA, healthy sleep score with overall cancer and specific cancer risk, and considering diverse genetic susceptibilities. In conclusion, the present study demonstrated that poor sleep score, low TPA level, and high SB level was independently associated with the risk of overall cancer as well as site-specific cancers. Moreover, low TPA-poor sleep group and high SB-poor sleep group had the highest risk for overall cancer, breast cancer, and lung cancer. Adherence to a healthier activity pattern was associated with a lower risk of cancer among individuals regardless of genetic strata. Our findings suggest that improving the quality of sleep and developing PA habits might yield benefits in mitigating the cancer risk. Further studies and clinical intervention trials are warranted to clarify the likely synergistic effects and the underlying mechanisms.

## STAR★Methods

### Key resources table

**Table:** 

REAGENT or RESOURCE	SOURCE	IDENTIFIER
**Deposited data**		

UK Biobank	https://biobank.ndph.ox.ac.uk/showcase/index.cgi	Application number: 66354

**Software and algorithms**		

R studio version 4.1.3	R software	https://www.rstudio.com/tags/rstudio/

### Resource availability

#### Lead contact

Further information and requests for resources should be directed to and will be fulfilled by the lead contact, Yuan Ding (dingyuan@zju.edu.cn).

#### Materials availability

This study did not generate new unique reagents.

#### Data and code availability

Consent given by the participants does not open for storage of data on an individual level in repositories or journals. Researchers who want access to datasets for replication should apply to UK Biobank directly (https://biobank.ndph.ox.ac.uk/showcase/index.cgi). The code used to generate the study database and perform statistical analyses could be applied by contacting the first author of this study, Rongqi Zhang (22118865@zju.edu.cn). Any additional information required to reanalyze the data reported in this paper is available from the lead contact upon request, Yuan Ding (dingyuan@zju.edu.cn).

### Experimental model and study participant details

This is a prospective study based on data from UKB, a large-scale cohort with 502,411 individuals aged 37–73 years old recruited from 22 centers in England, Scotland, and Wales between 2006 and 2010. The details of the UKB’s study design and methods have been described elsewhere.^38^ In brief, participants provided health-related data through touchscreen questionnaires, biological samples and physical examination. From 2009 to 2012, a total of 210,962 participants completed at least one 24-h dietary recall of the previous day. UKB has approval from the North West Multicenter Research Ethical Committee, and all participants have provided written informed consent. In this study, we excluded participants who had the history of cancer at baseline (*n* = 38,321) or had missing or unusable data for five sleep and circadian behaviors (*n* = 83,775), TPA (*n* = 19,093) or SB (*n* = 951). Finally, a total of 360,271 eligible participants free of cancer were included in this study.

### Method details

#### Assessment of sleep, physical activity, and sedentary behavior

We included five self-reported sleep and circadian behaviors, including duration, daytime napping, chronotype, insomnia symptom, and snoring. Information on these sleep traits was obtained from the touchscreen questionnaire. Those who responded ‘do not know’ or ‘prefer not to answer’ were excluded from the analysis. Morning chronotype or morning than evening chronotype, recommended sleep duration (7–8 h/d), never or rarely insomnia, no snoring, and no frequent daytime sleepiness, represented healthy sleep characteristics. The detailed definition of each item has been provided in Table S1. We assigned 1 or 0 score for each healthy or unhealthy sleep characteristic. Although the simple additive method constructing the composited sleep score had been used widely,^18^^,^^39^ the associations between each sleep trait and cancer risk were different. Therefore, we generated a weight sleep score by using the equation: weighted sleep score = (β_1_ × score of sleep duration + β_2_ × score of daytime napping + β_3_ × score of chronotype + β_4_ × score of insomnia + β_5_ × score of snoring)/(sum of the β coefficients).^39^ The weighted sleep score was then grouped into tertiles and divided into three categories: healthy (T3), intermediate (T2), and poor (T1).

Self-reported level of SB was measured via the touchscreen questionnaire. We summed the time of watching TV and using computer to assess the time of leisure-time SB and grouped it by tertile. The lower boundary of the second tertile was 3 h/d and the lower boundary of the third tertile was 4 h/d. Self-reported level of PA was qualified using the International Physical Activity Questionnaire (IPAQ).^40^ Based on the WHO guideline,^8^ PA was categorized as light (LPA, <3 MET), moderate (MPA, 3–6 MET), and vigorous (VPA, ≥6 MET). We calculated the time spent on each physical activity by multiplying the MET value of activity (3.3 METs for LPA, 4.0 METs for MPA, and 8.0 METs for VPA) by the time of PA (minutes per week)^41^ and summed the time in each category of physical activity to assess the total physical activity (TPA). Then TPA was grouped into tertiles the lower boundary of the second tertile was 971 MET min/week and the lower boundary of the third tertile was 2600 MET min/week.

#### Ascertainment of overall and site-specific cancer incidence

Data on cancer diagnosis were ascertained using a combination of records from the NHS Digital (cancer registry) and Public Health England for participants from England and Wales, NHS Central Register for participants from Scotland as well as the Hospital Episodes Statistics (HES) data for English participants and Scottish Morbidity Records (SMR) for Scottish participants. According to the World Health Organization’s ICD-10 codes, participants indicated as being a case if they had an incident diagnosis of cancer recorded as overall cancer (C00-97 excluding non-melanoma skin cancer: C44), colorectal cancer (C18-C20), breast cancer (C50), prostate cancer (C61), or lung cancer (C34) (Table S2). The first date of cancer incidence from the data linkage source was used as the diagnosis date.

#### Polygenic risk score for overall cancer

The genotyping process and arrays used in the UKB study have been described elsewhere.^42^ In the present study, we selected 597 independent SNPs as instrumental variables that were strongly associated with a total of 17 specific cancer types susceptibility (*p* < 5 × 10^−8^, r^2^ < 0.01) from the latest GWASs.^43^^,^^44^^,^^45^^,^^46^^,^^47^^,^^48^^,^^49^^,^^50^^,^^51^^,^^52^^,^^53^^,^^54^^,^^55^^,^^56^ Detailed information on these genetic instruments is provided in Table S3. Based on the selected SNPs, site-specific cancer polygenic risk scores (sPRS) were constructed for each participant by summing up the number of risk-increasing alleles for each SNP weighted by effect size on genetic liability to each specific cancer ($sPRS=\sum_{i=1}^{n}\beta i\times SNPi$). Overall cancer polygenic risk scores (oPRS) were calculated using a weighted method^57^ by estimating a weighted sPRS based on the standardized cancer incidence rate for each specific cancer in UKB ($oPRS=\sum_{i=1}^{k}hk\times sPRSi,k$) (Where the score is the overall cancer polygenic risk score of i^th^ individual, h_k_ is the age-standardized incidence of site-specific cancer k in the UK population, and sPRS_i,k_ is the aforementioned sPRS of site-specific cancer k). Then oPRS was grouped into quintiles and categorized into low (the lowest quintile), intermediate (quintiles 2–4), and high (highest quintile) genetic strata as previous study described.^58^

#### Definition of covariates

To reduce the impact of potential confounding, socio-demographic characteristics, lifestyle factors, and health status at baseline were selected as covariates based on published literature.^59^ We included age at recruitment (continuous), sex (male/female), ethnicity (white/non-white/unknown), education (college/high school and below/unknown), TDI (continuous), alcohol intake (never/former/current), height (continuous), body mass index (continuous), use of NSAIDs (yes/no), family history of cancer (yes/no), diabetes (yes/no), hypertension (yes/no), CVD (yes/no), age at menarche (continuous), number of live birth (continuous), use of hormones (yes/no) and use of oral contraceptive (yes/no), colorectal cancer screening (yes/no).

### Quantification and statistical analysis

Baseline characteristics of participants were described as means and standard deviations (SDs) for continuous variables, numbers (N) and percentages (%) for categorical variables stratified by the cancer status. Kaplan-Meier estimation, the scaled Schoenfeld residuals, and supremum tests of functional forms were used to examine the proportional hazards assumption, although no noticeable violations were observed. Kolmogorov-Smirnov test and homogeneity of variance test were conducted and variance inflation factor was calculated to examine the linearity assumption when sleep score was modeled continuously. Similarly, no noticeable violations were observed.

We tested the independent associations of five sleep traits, three categories of healthy sleep scores (poor, intermediate, healthy), three categories of TPA (low, intermediate, and high), and three categories of leisure-time SB (low, intermediate and high) with overall cancer and four site-specific cancers (prostate cancer, breast cancer, colorectal cancer, and lung cancer) risk by estimating the hazard ratio (HR) and 95% confidence intervals (CIs) using Cox-proportional hazard models with fully adjustment. For overall cancer, adjusting by age at recruitment (continuous), sex (male/female), ethnicity (white/non-white/unknown), education (college/high school and below/unknown), TDI (continuous), smoke (pack-year), alcohol intake (never/former/current), height (continuous), body mass index (continuous), use of NSAIDs (yes/no), family history of cancer (yes/no), diabetes (yes/no), hypertension (yes/no), CVD (yes/no). For breast cancer, additionally adjusting by age at menarche (continuous), number of live birth (continuous), use of hormones (yes/no) and use of oral contraceptive (yes/no). For colorectal cancer, additionally adjusting by colorectal cancer screening (yes/no). Then we tested the joint association of physical activity, sedentary behavior, and healthy sleep scores with cancer risk. And we calculated the *P*-interaction by conducting likelihood ratio test (LR-test) comparing a model with the interaction term to a model without. We further examined the joint effects of each activity and genetic susceptibility using Cox proportional hazard models and tested the interaction with genetic predisposition using LR-test.

Several sensitivity analyses were conducted to examine the robustness of our primary findings: (1) additionally adjusting for healthy diet score (continuous) and psychological health (yes/no) in Cox proportional hazards models, (2) further excluding cases occurred in the first two years of follow-up to avoid reverse causation, (3) further excluding those without any moderate-to-vigorous physical activity (MVPA) to avoid bias from extreme observations.

R version 4.1.3 was used to conduct data cleaning and statistical analyses. The statistical significance was defined as the two-side *p*-values < 0.05.

## Supplemental information

Document S1. Figure S1, Tables S1, S2 and S4–S8

Table S3. SNP list used in PRS
