# Supplementary material for: Sleep, physical activity, and sedentary behaviors in relation to overall cancer and site-specific cancer risk: A prospective cohort study
Source: iScience. 2024 May 9;27(6):109931. doi: 10.1016/j.isci.2024.109931 (PMC11225818; doi:10.1016/j.isci.2024.109931)
Supplement: Document S1. Figure S1, Tables S1, S2 and S4–S8 [file mmc1.pdf]

iScience, Volume *27*

Supplemental information

Sleep, physical activity, and sedentary behaviors in relation to overall cancer and site-specific cancer risk: A prospective cohort study

Rongqi Zhang, Ying Lu, Zilong Bian, Siyun Zhou, Liying Xu, Fangyuan Jiang, Shuai Yuan, Xiao Tan, Xiangjun Chen, Yuan Ding, and Xue Li

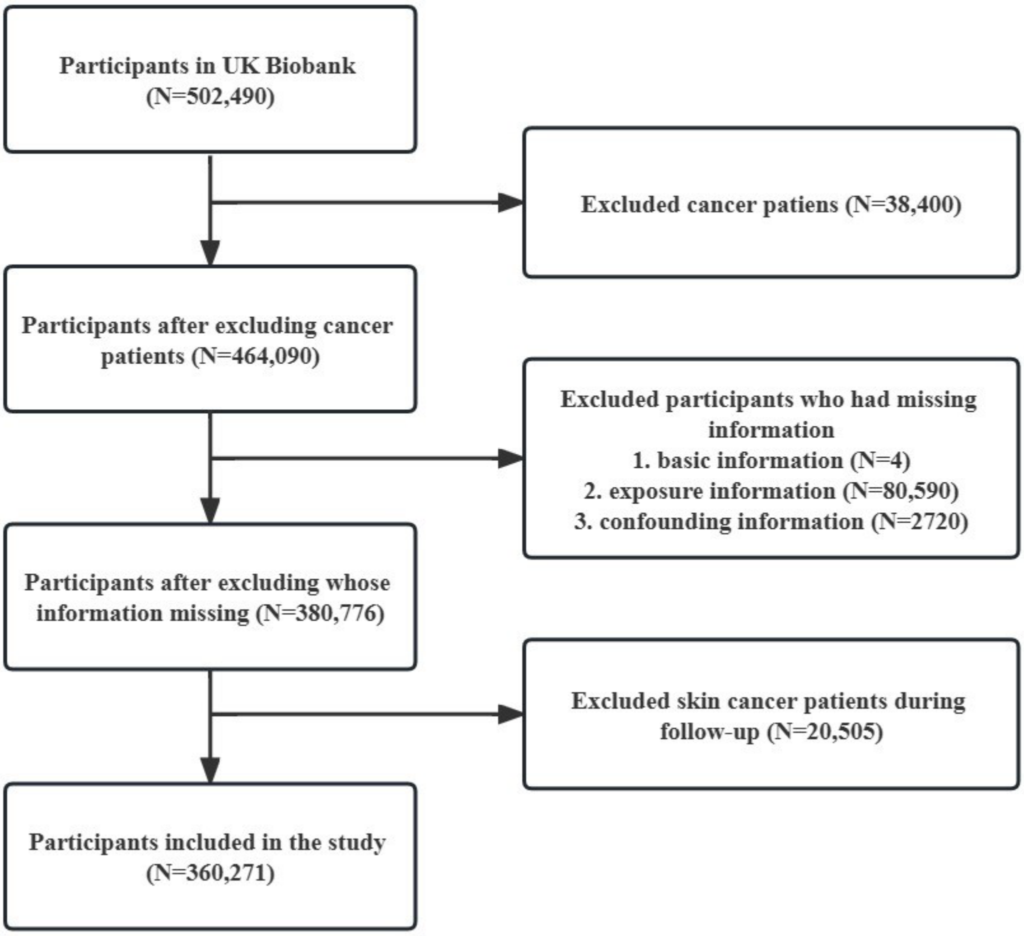

Supplementary Figure 1 Flowchart of the participants selection

Supplementary Table 1 The Scoring System of Sleep

Characteristics	UKB Code	UKB Questionnaire	Healthy	Unhealthy
Chronotype	1180	Do you consider yourself to be?	Morning or morning than evening	Evening than evening or morning
Sleep Duration	1160	About how many hours sleep do you get in every 24 hours? (Please include naps)	7-8 hours/day	<7 or >8 hours/day
Insomnia	1200	Do you have trouble falling asleep at night or do you wake up in the middle of the night?	Never/rarely	Sometimes or usually
Snoring	1210	Does your partner or a close relative or friend complain about your snoring?	No	Yes
Daytime napping	1220	How likely are you to doze off or fall asleep during the daytime when you don't mean to? (e.g., when working, reading or driving)	Never or sometimes	Often or all of the time

Supplementary Table 2 The Definition of Outcomes

Cancer site/cancer	Definition
Overall cancers	The definition of total cancer excludes in situ, benign, uncertain, melanoma, skin cancer or non-well-defined cancers. The ICD-10 code beginning with ('C0' 'C1' 'C2' 'C3' 'C4' 'C5' 'C6' 'C70' 'C71' 'C72' 'C73' 'C74' 'C75' 'C7A' 'C8' 'C9') or the ICD-9 code within (140.0-194.9/ 199.0-209.3) were included.
Colorectal cancer	C18.X, C19.X, C20.X
Lung cancer	C34.X
Prostate cancer	C61.X
Breast cancer	C50.X

Supplementary Table 4 The associations of each sleep trait with overall cancer and specific cancer risk

Sleep trait	Overall cancer[1]			Specific cancer											
				Prostate cancer			Lung cancer			Colorectal cancer[2]			Breast cancer[3]		
	N (case)	HR (95%CI)	*P* value	N (case)	HR (95%CI)	*P* value	N (case)	HR (95%CI)	*P* value	N (case)	HR (95%CI)	*P* value	N (case)	HR (95%CI)	*P* value
Sleep duration															
7-8 h/d (n=246,004)	27,786	Ref.		5,987	Ref.		5,476	Ref.		3,075	Ref.		1,711	Ref.	
<7 h/d (n=87,510)	9,940	0.98 (0.96-1.00)	0.080	1,959	0.98 (0.93-1.03)	0.475	1,720	1.04 (0.95-1.14)	0.393	1,080	1.01 (0.94-1.08)	0.854	761	0.91 (0.86-0.96)	**7.71E-04**
>8 h/d (n=26,757)	3,671	1.03 (0.99-1.07)	0.109	698	0.93 (0.86-1.01)	0.069	621	1.04 (0.92-1.18)	0.511	405	1.03 (0.92-1.14)	0.630	293	0.97 (0.89-1.05)	0.424
Per hour (n=360,271)	41,397	1.02 (1.01-1.03)	**2.77E-04**	8,644	1.00 (0.98-1.02)	0.781	7,817	0.99 (0.96, 1.02)	0.606	4,560	1.01 (0.98, 1.04)	0.451	2,765	1.03 (1.00-1.05)	**2.01E-02**
Daytime napping															
Never (n=275,196)	30,417	Ref.		6,157	Ref.		6,043	Ref.		3,339	Ref.		1,968	Ref.	
Sometimes (n=75,133)	9,635	0.99 (0.97-1.01)	0.395	2,200	1.00 (0.96-1.06)	0.866	1,565	0.95 (0.87-1.03)	0.226	1,078	1.00 (0.93-1.07)	0.913	691	1.01 (0.95-1.07)	0.829
Often (n=9942)	1,345	1.02 (0.97-1.08)	0.422	287	1.01 (0.89-1.13)	0.919	209	0.84 (0.69-1.03)	0.089	143	1.01 (0.85-1.20)	0.895	106	1.07 (0.93-1.24)	0.319
Chronotype															
Morning (n=97,269)	11,424	Ref.		2,422	Ref.		2,014	Ref.		1,278	Ref.		725	Ref.	
Morning than evening (n=127,693)	14,451	1.00 (0.98-1.03)	0.994	3,142	0.97 (0.92-1.03)	0.328	2,767	1.02 (0.92-1.13)	0.676	1,602	0.98 (0.91-1.06)	0.669	848	1.04 (0.99-1.11)	0.143

Evening than morning (n=102,904)	11,738	1.04 (1.02-1.07)	**1.19E-03**	2,389	1.00 (0.95-1.06)	0.933	2,328	1.26 (1.14-1.39)	**8.64E-06**	1,292	1.04 (0.96-1.12)	0.377	844	1.12 (1.06-1.19)	**2.12E-04**
Evening (n=32,405)	3,784	1.09 (1.05-1.13)	**8.15E-06**	691	0.98 (0.90-1.06)	0.601	708	1.61 (1.42-1.84)	**4.95E-13**	388	1.02 (0.91-1.15)	0.684	348	1.14 (1.04-1.24)	**3.95E-03**
Insomnia															
Never/rarely (n=88,844)	9,583	Ref.		2,468	Ref.		1,472	Ref.		1,085	Ref.		567	Ref.	
Sometimes (n=172,140)	19,648	1.02 (0.99-1.04)	0.175	3,965	1.05 (1.00-1.10)	0.080	3,843	1.02 (0.92-1.13)	0.701	2,234	1.05 (0.97-1.13)	0.223	1,292	1.00 (0.94-1.07)	0.917
Usually (n=99,287)	12,166	1.04 (1.01-1.07)	**3.80E-03**	2,211	1.11 (1.04-1.17)	**9.40E-04**	2,502	1.00 (0.89-1.11)	0.945	1,241	0.98 (0.90-1.07)	0.615	906	1.04 (0.98-1.12)	0.219
Snoring															
No (n=225,982)	24,844	Ref.		4,441	Ref.		5,403	Ref.		2,656	Ref.		1,683	Ref.	
Yes(n=134,289)	16,553	1.03 (1.01-1.05)	**5.27E-03**	4,203	1.04 (1.00-1.09)	**4.90E-02**	2,414	1.10 (1.02-1.19)	**1.84E-02**	1,904	1.07 (1.01-1.14)	**2.64E-02**	1,082	1.07 (1.02-1.13)	**4.78E-03**

The value in bold denotes significant differences ($p < 0.05$).

[1] Adjusted by age at recruitment (continuous), sex (male/female), ethnicity (white/non-white/unknown), education (college/high school and below/ unknown), TDI (continuous), smoke (pack-year), alcohol intake (never/former/current), height (continuous), body mass index (continuous), use of NSAIDs (yes/no), family history of cancer (yes/no), diabetes (yes/no), hypertension (yes/no), CVD (yes/no). Different sleep traits mutually adjusted.

[2] Additionally adjusted by colorectal cancer screening (yes/no).

[3] Additionally adjusted by age at menarche (continuous), number of live birth (continuous), use of hormones (yes/no) and use of oral contraceptive (yes/no).

Supplementary Table 5 Sensitivity analysis: additional adjustment of healthy diet score and psychological health.

Activity[1]	Overall cancer [2]			Specific cancer risk											
				Prostate cancer			Breast cancer[3]			Colorectal cancer[4]			Lung cancer		
	N (case)	HR (95%CI)	P value	N (case)	HR (95%CI)	P value	N (case)	HR (95%CI)	P value	N (case)	HR (95%CI)	P value	N (case)	HR (95%CI)	P value
Sleep score															
Healthy	8,234	Ref.		1632	Ref.		1568	Ref.		917	Ref.		523	Ref.	
Intermediate	18,845	1.02 (0.99-1.05)	0.160	4180	1.06 (1.00-1.13)	**3.75E-02**	3496	1.04 (0.98-1.11)	0.232	2105	1.00 (0.92-1.08)	0.980	1118	1.04 (0.93-1.16)	0.470
Poor	14,318	1.07 (1.04-1.10)	**3.66E-06**	2832	1.07 (1.01-1.14)	**2.79E-02**	2753	1.13 (1.06-1.21)	**2.24E-04**	1538	1.03 (0.95-1.12)	0.468	1124	1.29 (1.15-1.44)	**7.21E-06**
Total physical activity															
High	13,115	Ref.		3036	Ref.		2169	Ref.		1442	Ref.		859	Ref.	
Medium	13,992	1.01 (0.98-1.03)	0.634	2963	1.01 (0.96-1.07)	0.592	2801	1.07 (1.01-1.14)	**1.60E-02**	1541	1.03 (0.96-1.11)	0.395	827	0.92 (0.83-1.02)	0.107
Low	14,290	1.04 (1.02-1.07)	**1.50E-03**	2645	1.01 (0.95-1.06)	0.849	2847	1.09 (1.03-1.16)	**4.17E-03**	1577	1.07 (1.00-1.16)	0.063	1079	1.10 (1.00-1.22)	**4.48E-02**
Sedentary behavior															
Low	10,480	Ref.		1994	Ref.		2465	Ref.		1127	Ref.		560	Ref.	
Medium	15,977	1.03 (1.00-1.05)	0.060	3437	1.03 (0.98-1.09)	0.280	3043	1.02 (0.97-1.08)	0.435	1753	1.03 (0.95-1.13)	0.462	985	1.05 (0.94-1.17)	0.393
High	14,940	1.04 (1.02-1.07)	**1.64E-03**	3213	1.01 (0.95-1.07)	0.706	2309	1.00 (0.94-1.06)	0.892	1680	1.10 (0.95-1.26)	0.202	1220	1.21 (1.09-1.36)	**5.73E-04**

The value in bold denotes significant differences ($p < 0.05$).

[1] Sleep scores were categorized into: poor, T1; intermediate, T2; healthy, T3. Total physical activity was grouped as low level <971.000 MET min/week; medium level 971.000-2600.565 MET min/week; high level >2600.565 MET min/week. Sedentary behavior was grouped as low level <3 h/day; medium level 3-4 h/day; high level >4 h/day.

[2] Adjusted by age at recruitment (continuous), sex (male/female), ethnicity (white/non-white/unknown), education (college/high school and below/ unknown), TDI (continuous), smoke (pack-year), alcohol intake (never/former/current), height (continuous), body mass index (continuous), use of NSAIDs (yes/no), family history of cancer (yes/no), diabetes (yes/no), hypertension (yes/no), CVD (yes/no), healthy diet score (continuous) and psychological health (yes/no). Sleep score, physical activity and sedentary behavior were adjusted mutually.

[3] Additionally adjusted by colorectal cancer screening (yes/no).

[4] Additionally adjusted by age at menarche (continuous), number of live birth (continuous), use of hormones (yes/no) and use of oral contraceptive (yes/no).

Supplementary Table 6 Sensitivity analysis: excluding cases occurred in the first two years of follow-up.

Activity[1]	Overall cancer risk[2]			Specific cancer risk											
				Prostate cancer			Breast cancer[3]			Colorectal cancer[4]			Lung cancer		
	N (case)	HR (95%CI)	P value	N (case)	HR (95%CI)	P value	N (case)	HR (95%CI)	P value	N (case)	HR (95%CI)	P value	N (case)	HR (95%CI)	P value
Sleep score															
Healthy	7,122	Ref.		1,431	Ref.		1,324	Ref.		789	Ref.		461	Ref.	
Intermediate	16,298	1.02 (0.99-1.04)	0.285	3,629	1.05 (0.99-1.12)	0.110	2,956	1.04 (0.97-1.11)	0.277	1,810	1.01 (0.93-1.10)	0.861	988	1.03 (0.92-1.15)	0.572
Poor	12,371	1.07 (1.04-1.10)	**6.77E-06**	2,468	1.07 (1.00-1.14)	**5.11E-02**	2,284	1.10 (1.03-1.18)	**4.97E-03**	1,322	1.04 (0.95-1.14)	0.364	993	1.32 (1.18-1.48)	**8.12E-07**
Total physical activity															
High	12,275	Ref.		2,286	Ref.		2,370	Ref.		1,342	Ref.		937	Ref.	
Medium	12,168	1.01 (0.99-1.04)	0.290	2,594	1.01 (0.96-1.07)	0.701	2,383	1.11 (1.04-1.18)	**8.63E-04**	1,323	1.00 (0.93-1.08)	0.987	738	0.94 (0.85-1.04)	0.207
Low	11,348	1.04 (1.01-1.07)	**2.21E-03**	2,648	0.98 (0.93-1.04)	0.589	1,811	1.12 (1.05-1.19)	**6.04E-04**	1,256	1.04 (0.96-1.12)	0.329	767	1.11 (1.00-1.22)	**4.22E-02**
Sedentary behavior															
Low	9,107	Ref.		1,747	Ref.		2,080	Ref.		994	Ref.		487	Ref.	
Medium	13,820	1.03 (1.00-1.06)	0.049	3,003	1.04 (0.98-1.1)	0.226	2,527	1.02 (0.96-1.08)	0.469	1,486	1.00 (0.92-1.08)	0.933	880	1.10 (0.98-1.23)	0.093
High	12,864	1.05 (1.02-1.08)	**3.93E-04**	2,778	1.01 (0.95-1.08)	0.710	1,957	1.03 (0.97-1.10)	**3.22E-01**	1,441	1.07 (0.98-1.16)	0.126	1,075	1.28 (1.15-1.43)	**1.30E-05**

[1] Sleep scores were categorized into: poor, T1; intermediate, T2; healthy, T3. Total physical activity was grouped as low level <971.000 MET min/week; medium level 971.000-2600.565 MET min/week; high level >2600.565 MET min/week. Sedentary behavior was grouped as low level <3 h/day; medium level 3-4 h/day; high level >4 h/day.

[2] Adjusted by age at recruitment (continuous), sex (male/female), ethnicity (white/non-white/unknown), education (college/high school and below/ unknown), TDI (continuous), smoke (pack-year), alcohol intake (never/former/current), height (continuous), body mass index (continuous), use of NSAIDs (yes/no), family history of cancer (yes/no), diabetes (yes/no), hypertension (yes/no), CVD (yes/no). Sleep score, physical activity and sedentary behavior were adjusted mutually.

[3] Additionally adjusted by colorectal cancer screening (yes/no).

[4] Additionally adjusted by age at menarche (continuous), number of live birth (continuous), use of hormones (yes/no) and use of oral contraceptive (yes/no).

Supplementary Table 7 Sensitivity analysis: excluding participants without any moderate-to-vigorous physical activity.

Activity[1]	Overall cancer risk[2]			Specific cancer risk											
				Prostate cancer			Breast cancer[3]			Colorectal cancer[4]			Lung cancer		
	N (case)	HR (95%CI)	P value	N (case)	HR (95%CI)	P value	N (case)	HR (95%CI)	P value	N (case)	HR (95%CI)	P value	N (case)	HR (95%CI)	P value
Sleep score															
Healthy	6,945	Ref.		1,414	Ref.		1,337	Ref.		778	Ref.		414	Ref.	
Intermediate	15,990	1.02 (0.99-1.05)	0.189	3,662	1.07 (1.01-1.14)	**2.88E-02**	2,916	1.01 (0.95-1.08)	0.708	1,784	1.00 (0.92-1.09)	0.910	913	1.06 (0.94-1.19)	0.345
Poor	11,556	1.08 (1.04-1.11)	**1.88E-06**	2,415	1.10 (1.03-1.18)	**3.47E-03**	2,206	1.11 (1.03-1.18)	**4.19E-03**	1,243	1.04 (0.95-1.14)	0.364	840	1.33 (1.19-1.50)	**1.91E-06**
Total physical activity															
High	8,538	Ref.		1,715	Ref.		1,694	Ref.		959	Ref.		572	Ref.	
Medium	13,092	1.01 (0.98-1.03)	0.649	2,788	1.01 (0.96-1.06)	0.830	2,644	1.09 (1.03-1.16)	**2.49E-03**	1,436	1.01 (0.94-1.09)	0.761	758	0.93 (0.84-1.03)	0.153
Low	12,861	1.04 (1.01-1.07)	**4.38E-03**	2,988	1.02 (0.96-1.08)	0.584	2,121	1.10 (1.03-1.17)	**3.61E-03**	1,410	1.08 (0.99-1.17)	0.082	837	1.08 (0.97-1.20)	0.182
Sedentary behavior															
Low	9,054	Ref.		1,773	Ref.		2,101	Ref.		985	Ref.		458	Ref.	
Medium	13,565	1.03 (1.00-1.05)	0.057	3,044	1.03 (0.97-1.10)	0.269	2,545	1.04 (0.98-1.10)	0.246	1,475	1.00 (0.93-1.09)	0.911	812	1.10 (0.98-1.24)	0.103
High	11,872	1.05 (1.02-1.08)	**1.21E-03**	2,674	1.00 (0.94-1.07)	0.952	1,813	1.02 (0.95-1.09)	0.613	1,345	1.07 (0.98-1.17)	0.125	897	1.25 (1.12-1.41)	**1.57E-04**

[1] Sleep scores were categorized into: poor, T1; intermediate, T2; healthy, T3. Total physical activity was grouped as low level <971.000 MET min/week; medium level 971.000-2600.565 MET min/week; high level >2600.565 MET min/week. Sedentary behavior was grouped as low level <3 h/day; medium level 3-4 h/day; high level >4 h/day.

[2] Adjusted by age at recruitment (continuous), sex (male/female), ethnicity (white/non-white/unknown), education (college/high school and below/ unknown), TDI (continuous), smoke (pack-year), alcohol intake (never/former/current), height (continuous), body mass index (continuous), use of NSAIDs (yes/no), family history of cancer (yes/no), diabetes (yes/no), hypertension (yes/no), CVD (yes/no). Sleep score, physical activity and sedentary behavior were adjusted mutually.

[3] Additionally adjusted by colorectal cancer screening (yes/no).

[4] Additionally adjusted by age at menarche (continuous), number of live birth (continuous), use of hormones (yes/no) and use of oral contraceptive (yes/no).

Supplementary Table 8 STROBE Statement: checklist of items that should be included in reports of observational studies

	Item No	Recommendation
**Title and abstract**	1-2	(*a*) Indicate the study's design with a commonly used term in the title or the abstract
		(*b*) Provide in the abstract an informative and balanced summary of what was done and what was found
**Introduction**		
Background/rationale	3-4	Explain the scientific background and rationale for the investigation being reported
Objectives	5	State specific objectives, including any prespecified hypotheses
**Methods**		
Study design	24	Present key elements of study design early in the paper
Setting	24	Describe the setting, locations, and relevant dates, including periods of recruitment, exposure, follow-up, and data collection
Participants	24	(*a*) *Cohort study*—Give the eligibility criteria, and the sources and methods of selection of participants. Describe methods of follow-up<br>*Case-control study*—Give the eligibility criteria, and the sources and methods of case ascertainment and control selection. Give the rationale for the choice of cases and controls<br>*Cross-sectional study*—Give the eligibility criteria, and the sources and methods of selection of participants
		(*b*) *Cohort study*—For matched studies, give matching criteria and number of exposed and unexposed<br>*Case-control study*—For matched studies, give matching criteria and the number of controls per case
Variables	25-27	Clearly define all outcomes, exposures, predictors, potential confounders, and effect modifiers. Give diagnostic criteria, if applicable
Data sources/ measurement	24-26	For each variable of interest, give sources of data and details of methods of assessment (measurement). Describe comparability of assessment methods if there is more than one group
Bias	27	Describe any efforts to address potential sources of bias
Study size	24	Explain how the study size was arrived at
Quantitative variables	25-27	Explain how quantitative variables were handled in the analyses. If applicable, describe which groupings were chosen and why
Statistical methods	28-29	(*a*) Describe all statistical methods, including those used to control for confounding
		(*b*) Describe any methods used to examine subgroups and interactions
		(*c*) Explain how missing data were addressed
		(*d*) *Cohort study*—If applicable, explain how loss to follow-up was addressed<br>*Case-control study*—If applicable, explain how matching of cases and controls was addressed<br>*Cross-sectional study*—If applicable, describe analytical methods taking account of sampling strategy
		(*e*) Describe any sensitivity analyses

**Results**		
Participants	5*	(a) Report numbers of individuals at each stage of study—eg numbers potentially eligible, examined for eligibility, confirmed eligible, included in the study, completing follow-up, and analysed
		(b) Give reasons for non-participation at each stage
		(c) Consider use of a flow diagram
Descriptive data	5-6	(a) Give characteristics of study participants (eg demographic, clinical, social) and information on exposures and potential confounders
		(b) Indicate number of participants with missing data for each variable of interest
		(c) *Cohort study*—Summarise follow-up time (eg, average and total amount)
Outcome data	5	*Cohort study*—Report numbers of outcome events or summary measures over time
		Case-control study—Report numbers in each exposure category, or summary measures of exposure
		Cross-sectional study—Report numbers of outcome events or summary measures
Main results	6-7	(*a*) Give unadjusted estimates and, if applicable, confounder-adjusted estimates and their precision (eg, 95% confidence interval). Make clear which confounders were adjusted for and why they were included
		(*b*) Report category boundaries when continuous variables were categorized
		(*c*) If relevant, consider translating estimates of relative risk into absolute risk for a meaningful time period
Other analyses	7	Report other analyses done—eg analyses of subgroups and interactions, and sensitivity analyses
**Discussion**		
Key results	8	Summarise key results with reference to study objectives
Limitations	11-12	Discuss limitations of the study, taking into account sources of potential bias or imprecision. Discuss both direction and magnitude of any potential bias
Interpretation	9-11	Give a cautious overall interpretation of results considering objectives, limitations, multiplicity of analyses, results from similar studies, and other relevant evidence
Generalisability	11	Discuss the generalisability (external validity) of the study results
**Other information**		
Funding	14	Give the source of funding and the role of the funders for the present study and, if applicable, for the original study on which the present article is based

Note: An Explanation and Elaboration article discusses each checklist item and gives methodological background and published examples of transparent reporting. The STROBE checklist is best used in conjunction with this article (freely available on the Web sites of PLoS Medicine at http://www.plosmedicine.org/, Annals of Internal Medicine at http://www.annals.org/, and Epidemiology at http://www.epidem.com/). Information on the STROBE Initiative is available at www.strobe-statement.org.
